# Fetal hemodynamic changes and mitochondrial dysfunction in myocardium and brain tissues in response to anemia: a lesson from hemoglobin Bart’s disease

**DOI:** 10.1186/s12884-023-06232-x

**Published:** 2024-02-16

**Authors:** Suchaya Luewan, Nattayaporn Apaijai, Nipon Chattipakorn, Siriporn C. Chattipakorn, Theera Tongsong

**Affiliations:** 1 Department of Obstetrics and Gynecology, Division of Maternal Fetal Medicine, Chiang Mai University, Chiang Mai, Thailand; 2 Cardiac Electrophysiology Research and Training Center (CERT), Faculty of Medicine, Chiang Mai University, Chiang Mai, Thailand; 3 Cardiac Electrophysiology Unit, Department of Physiology, Faculty of Medicine, Chiang Mai University, Chiang Mai, Thailand; 4 Center of Excellence in Cardiac Electrophysiology Research, Chiang Mai University, Chiang Mai, Thailand; 5 Department of Oral Biology and Diagnostic Sciences, Faculty of Dentistry, Chiang Mai University, Chiang Mai, Thailand

**Keywords:** Anemia, Brain, Fetus, Heart, Hemodynamics, Hemoglobin Bart’s disease

## Abstract

**Objective:**

Whether or not the effects of anemia in the early phase, while the fetuses attempts to increase cardiac output to meet oxygen requirement in peripheral organs, is detrimental to the fetal developing vital organs is little-known. The objective of this is to compare prenatal cardiovascular changes and post-abortal cellular damages in the myocardium as a pumping organ and the brain as a perfused organ between anemic fetuses (using fetal Hb Bart’s disease as a study model) in pre-hydropic phase and non-anemic fetuses.

**Methods:**

Fetuses affected by Hb Bart’s disease and non-anemic fetuses at 16–22 weeks were recruited to undergo comprehensive fetal echocardiography. Cord blood analysis was used to confirm the definite diagnosis of fetal Hb Bart’s disease and normal fetuses. Fetal cardiac and brain tissues were collected shortly after pregnancy termination for the determination of oxidative stress and mitochondrial function, including mitochondrial ROS production and mitochondrial membrane changes.

**Results:**

A total of 18 fetuses affected by Hb Bart’s disease and 13 non-anemic fetuses were recruited. The clinical characteristics of both groups were comparable. The affected fetuses showed a significant increase in cardiac dimensions, cardiac function, cardiac output and brain circulation without deteriorating cardiac contractility and preload. However, in the affected fetuses, mitochondrial dysfunction was clearly demonstrated in brain tissues and in the myocardium, as indicated by a significant increase in the membrane potential change (p-value < 0.001), and a significant increase in ROS production in brain tissues, with a trend to increase in myocardium. The findings indicated cellular damage in spite of good clinical compensation.

**Conclusion:**

The new insight is that, in response to fetal anemia, fetal heart increases in size (dilatation) and function to increase cardiac output and blood flow velocity to provide adequate tissue perfusion, especially brain circulation. However, the myocardium and brain showed a significant increase in mitochondrial dysfunction, suggesting cellular damage secondary to anemic hypoxia. The compensatory increase in circulation could not completely prevent subtle brain and heart damage.

## Introduction

The impact of anemia on childhood and adult health has been extensively studied, leading to successful management. However, knowledge regarding its impact on fetal life is very limited, and the extent to which fetal anemia without treatment could negatively impact later life, especially heart health, has never been thoroughly explored. This is because of the several limitations associated with conducting a study on live fetuses in utero. Theoretically, anemic hypoxia tends to cause cellular damage that affects health in later life, as already known based on the concept of fetal origin of adult disease [1–4]. We can expect that several fetuses with non-lethal anemia due to any causes can have anemic hypoxia with some degree of cellular damages, but we have no solid evidence to clarify the association and the extent of the hypoxic injuries. Certainly, fetal anemia or low levels of hemoglobin causes a reduction in oxygen delivery in all target organs, resulting in tissue hypoxia and cellular damage in various vital organs. Theoretically, fetal anemic hypoxia secondary to various causes, like Rh parvovirus B19, alloimmunization or fetal alpha-thalassemia, plays a major role in adverse outcomes, including heart failure and hydropic changes due to hemodynamic disturbance. Fetal anemia can cause high output cardiac failure and hydrops fetalis. However, the effects of the early (pre-hydropic) phase of fetal anemia on molecular changes of cardiovascular system are little known. The main purpose of fetal response to anemia is to maintain oxygen delivery in the target organs by making the cardiovascular system to increase cardiac output. It is unclear whether or not overworking the heart to increase cardiac output to meet the requirement of end organ perfusion is detrimental to the fetal heart and whether or not target organs, such as the brain, are adequately perfused. It is theoretically possible that cellular damage in both the pumping organ (heart) and perfused organs (such as the brain) may have already occurred in the early phase of anemia before the appearance of clinical manifestations, like heart failure or hydrops fetalis. In fact, in our preliminary study, fetal anemia due to Hb Bart’s disease can cause abnormal mitochondrial function and cellular damage in the myocardium at the early stage of cardiac compensation [5]. Accordingly, this study was conducted to investigate the association between the increased work of the fetal cardiovascular system in response to fetal anemia (assessed by comprehensive fetal echocardiography) and cellular damage in the fetal myocardium and brain tissue. These subtle changes caused by anemia might adversely affect the developing heart and brain or may be associated with future cardiovascular issues in adult life, known as fetal programming.

The main objective of this study is to investigate whether fetal response to anemia is detrimental to the pumping machine (heart), as a consequence of increased work, and the target organ (represented by the fetal brain), as an indication of the effectiveness of compensation in providing oxygen delivery. This objective is achieved by comparing (1) cellular damages, assessed through mitochondrial function and oxidative stress levels in the fetal myocardium and brain tissue, and (2) clinical changes in the cardiovascular system, including cardiac function, morphology and cerebral blood flow, assessed by comprehensive fetal echocardiography, between anemic fetuses, using fetal hemoglobin (Hb) Bart’s disease as a study model, and non-anemic fetuses.

## Materials and methods

A cross-sectional (prospective) analytical study was undertaken at Maharaj Nakorn Chiang Mai Hospital (university hospital) from 1 January 2019 to 31 May 2022. The study was ethically approved by the Institutional Review Board (Ethics Committee 4; Research ID OBG-2564-088870). Pregnant women who had fetuses with Hb Bart’s disease at gestational age of 12–22 weeks were prospectively recruited, under our project of prenatal control of severe thalassemia [6]. The inclusion criteria for the study group are as follows: (1) Pregnant women with fetuses affected by Hb Bart’s disease, confirmed by cord blood analysis. (2) Gestational age of 16–22 weeks, based on fetal biometry in the first half of pregnancy (crown-rump length: CRL or biparietal diameter: BPD). (3) No hydropic signs on fetal ultrasound examination. Exclusion criteria are as follows: (1) Fetuses with chromosome abnormalities, growth abnormalities or structural anomalies other than Hb Bart’s disease. (2) Poor quality ultrasound images of Doppler studies. (3) Fetuses aborted at more than 48 h after ultrasound examination. (4) Maternal underlying medical disease. (5) parents preferred not to perform autopsy. To compare the mitochondrial function and oxidative stress levels, as well as prenatal cardiovascular changes, the control group was recruited from pregnant women with non-anemic fetuses, such as beta thalassemia hemoglobin E disease and homozygous beta thalassemia, who underwent pregnancy termination.

### Data collection

Pregnant women meeting the inclusion criteria were enrolled to participate in the study with written informed consent. The demographic data were collected, including maternal age, gravidity, gestational age at diagnosis and pregnancy termination, history of previous pregnancy, laboratory data at first antenatal care, thalassemia screening and diagnostic test as well as prenatal diagnostic procedure. Ultrasonography was performed within 24 h before termination of pregnancy, and the myocardium and brain tissue were collected after termination for evaluation of mitochondrial functioning. Concerning the terminated pregnancies, myocardium and brain tissues were collected, put in an icebox and immediately transferred to the laboratory station for cellular tests.

### Prenatal fetal echocardiography

Ultrasonographic evaluation was performed by the maternal-fetal medicine (MFM) research team after orientation to minimize inter-observer variation, using Voluson E8 or Voluson E10 (GE Medical Systems, Zipf, Austria) equipped with a transabdominal curvilinear transducer of frequency 3.5 to 5 MHz. In addition to standard ultrasound examination, comprehensive cardiovascular assessment was performed, including cardiac dimensions (diameter, area and cardio-thoracic diameter ratio), global sphericity index (GSI: cardiac length to cardiac width ratio), combined cardiac output, middle cerebral artery – peak systolic velocity (MCA-PSV), middle cerebral artery – pulsatility index (MCA-PI), myocardial performance index (MPI) as well as isovolumetric contraction time (ICT), isovolumetric relaxation time (IRT), ejection time (ET), shortening fraction (SF) of the ventricular contractility, and preload index in the ductus venosus (PLI). The techniques of fetal echocardiographic assessment used in this study are comprehensively described in our previous studies [7–13].

### Mitochondrial function study

#### Brain mitochondrial isolation

Immediately after heart and brain are removed from the miscarriage fetus, the brain tissue (frontal or parietal lobes) was homogenized according to the previous study [14]. Isolated brain mitochondria were used to determine mitochondrial function including brain mitochondrial reactive oxygen species (ROS) levels, brain mitochondrial membrane potential changes, and brain mitochondrial swelling.

#### Cardiac mitochondrial isolation

Immediately after heart is removed from the miscarriage fetus, the heart (left ventricle) is finely minced, and homogenized in ice-cold buffer containing (in mmol/l) sucrose 300, TES 5 and EGTA 0.2, pH 7.2. The homogenates are centrifuged at 800 g for 5 min and the supernatant is collected and centrifuged at 8,800 g for 5 min. The resulting mitochondrial pellet is resuspended in ice-cold buffer and centrifuged once more at 8,800 g for 5 min. Finally, the mitochondrial pellet is resuspended in a respiration buffer (containing 100 mM KCl, 50 mM sucrose, 10 mM HEPES, 5 mM KH2PO4, pH 7.4 at 25 ºC). Protein concentration is determined according to the bicinchoninic acid (BCA) assay [15].

#### Determination of cardiac and brain mitochondrial ROS production

ROS production is measured with the dye dichlorohydrofluorescein diacetate (DCFH-DA) [15]. Isolated cardiac and brain mitochondria are incubated with 2-µM DCFH-DA at 25 ºC for 20 min. ROS levels are determined via a fluorescent microplate reader at λex 485 nm and λem 530 nm. Moreover, 2 mM of hydrogen peroxide (H_2_O_2_) was added to mimic a severe oxidative stress condition, and the percentage changes of fluorescence intensity between mitochondria with and without H_2_O_2_ was calculated.

#### Determination of cardiac and brain mitochondrial membrane potential change

Mitochondrial membrane potential change (ΔΨm) is measured with the dye 5,5’,6,6’-tetrachloro-1,1’,3,3’-tetraethylbenzimidazolcarbocyanine iodide (JC-1) [15]. Isolated cardiac and brain mitochondria are stained with JC-1 (310 nM) at 37 ºC for 30 min. Mitochondrial membrane potential is determined as fluorescence intensity using of a fluorescent microplate reader. JC-1 monomer (green) fluorescence is excited at λex 485 nm and λem 530 nm. JC-1 aggregate form (red) fluorescence is λex 485 nm and λem 590 nm. The change in mitochondrial membrane potential is calculated as the ratio of red to green fluorescence. Mitochondrial depolarization is indicated by a decrease in the red/green fluorescence intensity ratio. Moreover, 2 mM of hydrogen peroxide (H_2_O_2_) was added to mimic a severe mitochondrial depolarization, and the percentage changes of fluorescence intensity between mitochondria with and without H_2_O_2_ was calculated.

### Statistical analysis

All statistical analyses were performed using the statistical package for the social sciences (SPSS) software, version 26.0 (IBM Corp. Released 2019. IBM SPSS Statistics for Windows, Version 26.0 Armonk, NY: IBM Corp). The baseline data were presented as mean ± SD or median (interquartile range) for continuous data, as appropriate for data distribution. For comparison of the categorical data, Chi-square was used, whereas Mann-Whitney-U and Student T tests were used for the comparison of continuous data. The statistically significant value was P < 0.05.

## Results

During the study period, a total of 80 pregnancies at risk of fetal Hb Bart’s disease were recruited to undergo comprehensive fetal cardiovascular ultrasound and invasive prenatal diagnosis. Of them, 18 cases were proven to be affected by Hb Bart’s diseases, and termination of pregnancy with post-abortal tissue sampling was performed. During the same period, a total of 13 normal pregnancies indicated for pregnancy termination were enrolled in the control group. The baseline characteristics of the participants were comparable in both groups (P-value > 0.05). The mean (+ SD) gestational age of the affected and control groups at prenatal diagnosis were 19.4 ± 2.7 and 19.6 ± 2.9 weeks, respectively (P-value 0.846), as presented in Table 1. Note that fetal hemoglobin and hematocrit were significantly lower in the group of Hb Bart’s disease (P-value < 0.001). The results of fetal cardiovascular ultrasound are presented in Table 1. Based on morphologic study, the cardiac size of the affected fetuses significantly increased both in diameter and area, with a greater increase in transverse diameter than long-axis, resulting in a significant decrease in GSI. Of note, despite cardiac enlargement, ventricular wall thickness was not significantly different from that of the control group. Based on cardiac function study, in the affected fetuses, the combined cardiac output significantly increased, whereas the shortening fraction of both sides and the ductus venosus preload index were comparable with those of the control group. Notably, in the affected group, the MPI of the right side significantly increased, whereas that of the left side had an increasing trend but did not reach statistical significance. Additionally, ICT of both sides were significantly higher in the affected group, as presented in Table 1. Other cardiac parameters were comparable between both groups. Based on cerebral blood flow study, MCA-PSV was significantly higher in the affected fetuses, with a significant decrease in MCA-PI, implying an increase in the end-diastolic flow.

**Table 1:** Clinical characteristics of the pregnancies with fetuses affected by hemoglobin Bart’s disease and those with non-anemic fetuses

Characteristics	Affected fetuses N:18	Non-anemic fetuses; N: 13	P-value*
Baseline characteristics			
Maternal age (years): mean ± SD	26.3 ± 4.5	28.8 ± 5.9	0.204
Parity; nulliparity : N (%)	13 (76.5%)	9 (69.2%)	0.657^#^
Gestational age at sampling (weeks): mean ± SD	19.4 ± 2.7	19.6 ± 2.9	0.846
Biparietal diameter (cm): mean ± SD	4.5 ± 0.8	4.6 ± 1.0	0.571
Hemoglobin (g/dl): mean ± SD	6.4 ± 1.3	11.2 ± 1.2	< 0.001
Hematocrit (%): mean ± SD	26.9 ± 5.6	37.1 ± 3.8	< 0.001
Cardiac morphology			
Cardio-thoracic diameter ratio : mean ± SD	0.60 ± 0.07	0.45 ± 0.04	< 0.001
Cardio-thoracic area ratio : mean ± SD	0.39 ± 0.07	0.26 ± 0.04	< 0.001
Left ventricular wall thickness (mm) : mean ± SD	1.77 ± 0.49	1.69 ± 0.45	0.643
Right ventricular wall thickness (mm) : mean ± SD	1.78 ± 0.45	1.57 ± 0.53	0.251
Global sphericity index (GSI): mean ± SD	1.07 ± 0.21	1.27 ± 0.12	0.005
Cardiac function			
Combined cardiac output Z-score: mean ± SD	1.55 ± 0.77	0.95 ± 0.48	0.020
Right myocardial performance index: mean ± SD	0.57 ± 0.13	0.48 ± 0.10	0.036
Right isovolumic contraction time: mean ± SD	45.44 ± 14.10	35.56 ± 10.31	0.041
Right isovolumic relaxation time: mean ± SD	48.50 ± 10.12	46.48 ± 7.47	0.545
Right ejection time: mean ± SD	154.56 ± 14.95	152.31 ± 13.69	0.827
Left myocardial performance index: mean ± SD	0.55 ± 0.13	0.49 ± 0.11	0.203
Left isovolumic contraction time: mean ± SD	48.44 ± 16.97	33.38 ± 11.99	0.010
Left isovolumic relaxation time: mean ± SD	44.39 ± 9.50	45.15 ± 7.41	0.811
Left ejection time: mean ± SD	157.67 ± 12.63	157.54 ± 13.18	0.839
Right shortening fraction (%): mean ± SD	35 ± 7	40 ± 11	0.173
Left shortening fraction (%): mean ± SD	39 ± 13	39 ± 9	0.816
Ductus venosus preload index: mean ± SD	0.50 ± 0.25	0.48 ± 0.15	0.786
Cerebral circulation			
MCA-PSV (cm/sec): mean ± SD	38.88 ± 8.05	26.97 ± 11.58	< 0.001
MCA-PSV (MoMs): mean ± SD	1.56 ± 0.52	0.85 ± 0.43	< 0.001
MCA-PI: mean ± SD	1.31 ± 0.26	1.51 ± 0.23	0.029

* Student T test; # Chi-square test

**Fig. 1:**
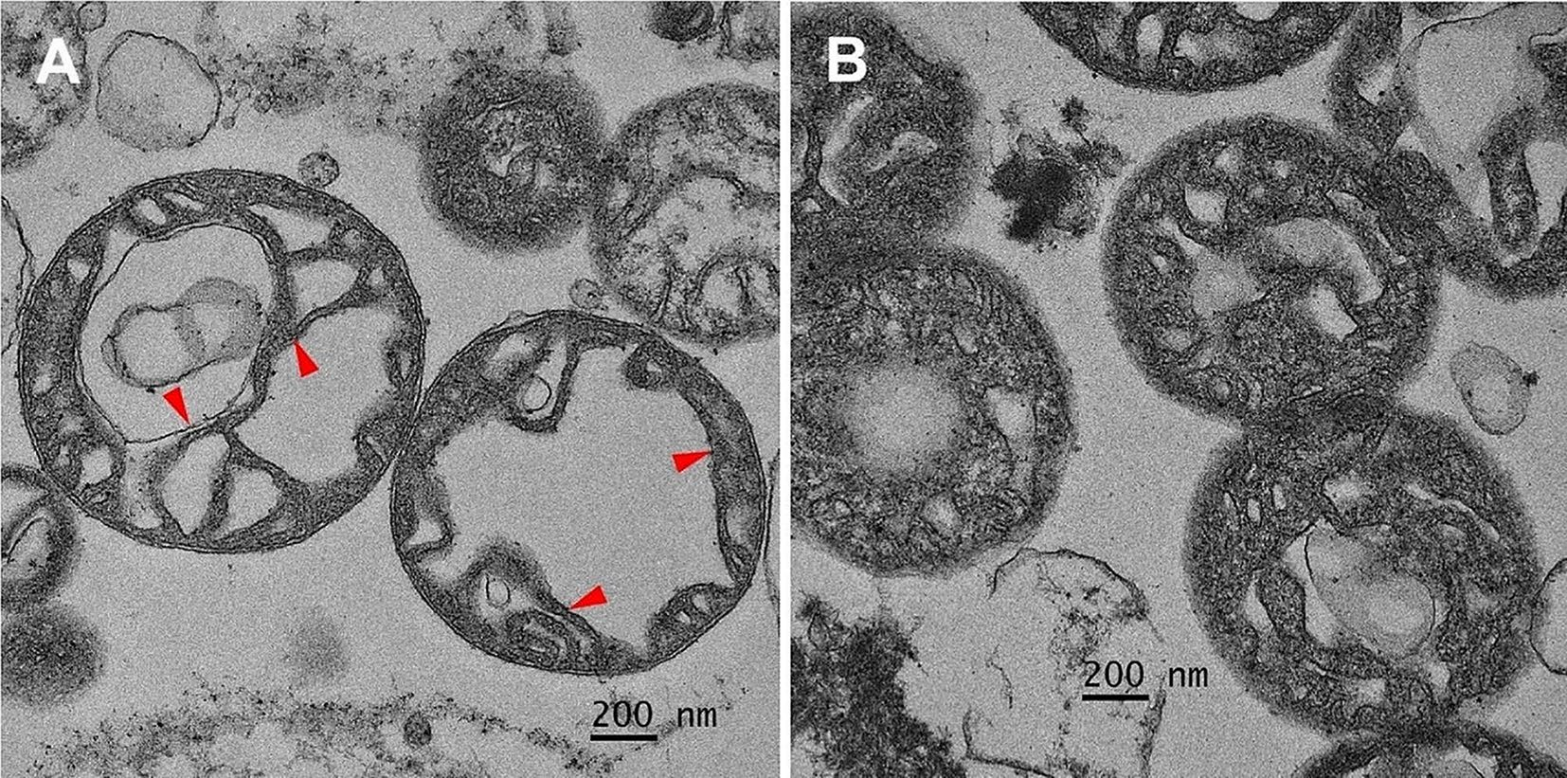
Electron micrographs of cardiac mitochondria demonstrate that the fetus affected by Hb Bart’s disease (**A**) had unfolded cristae (arrowhead), indicating cardiac mitochondrial swelling when compared with those of a non-anemic fetus (**B**)

**Fig. 2:**
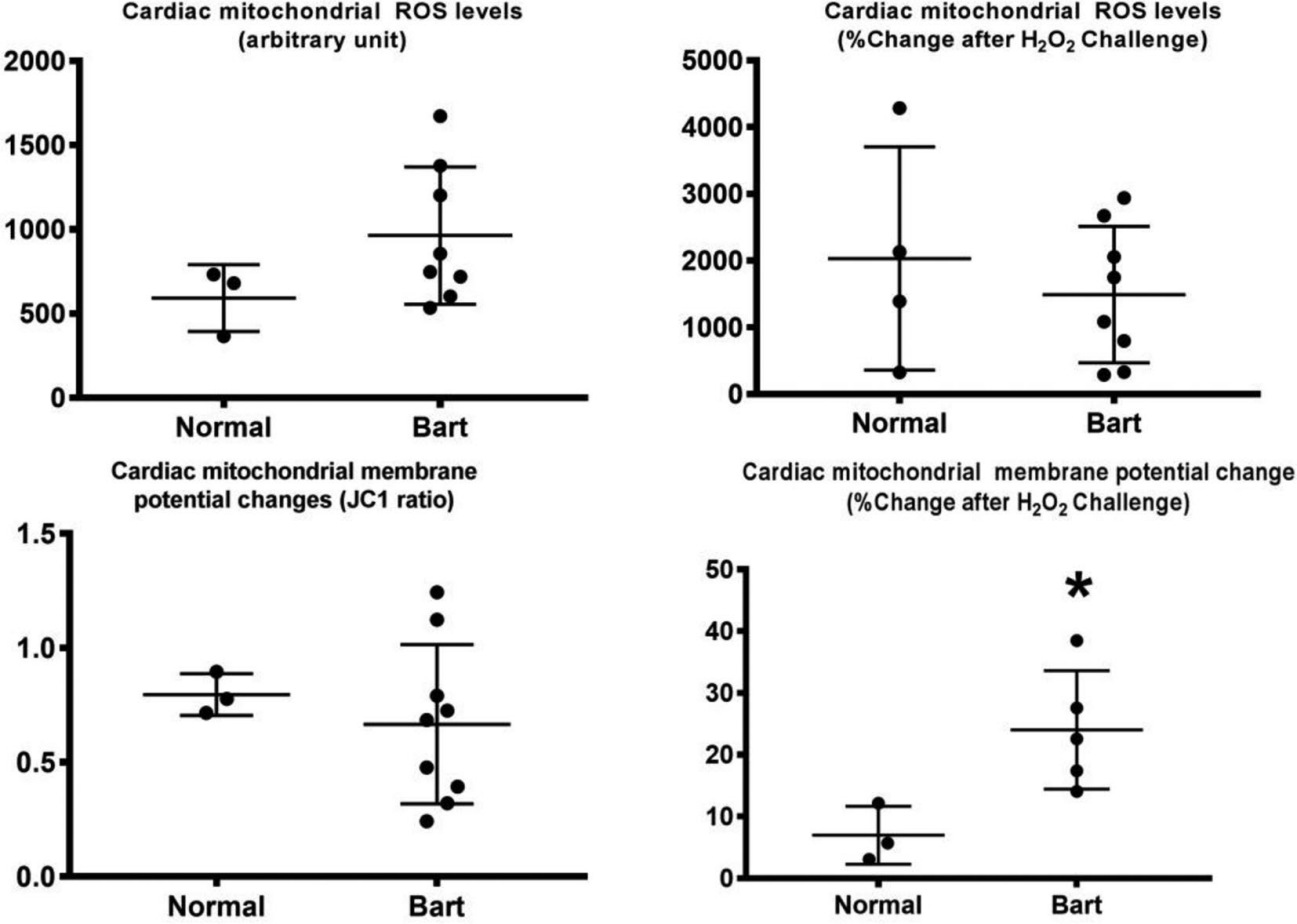
Mitochondrial dysfunction, as indicated by increased membrane potential change, was observed in the hearts of fetuses affected with Hb Bart’s disease compared with those of the normal ones (*p-value < 0.001)

**Fig. 3:**
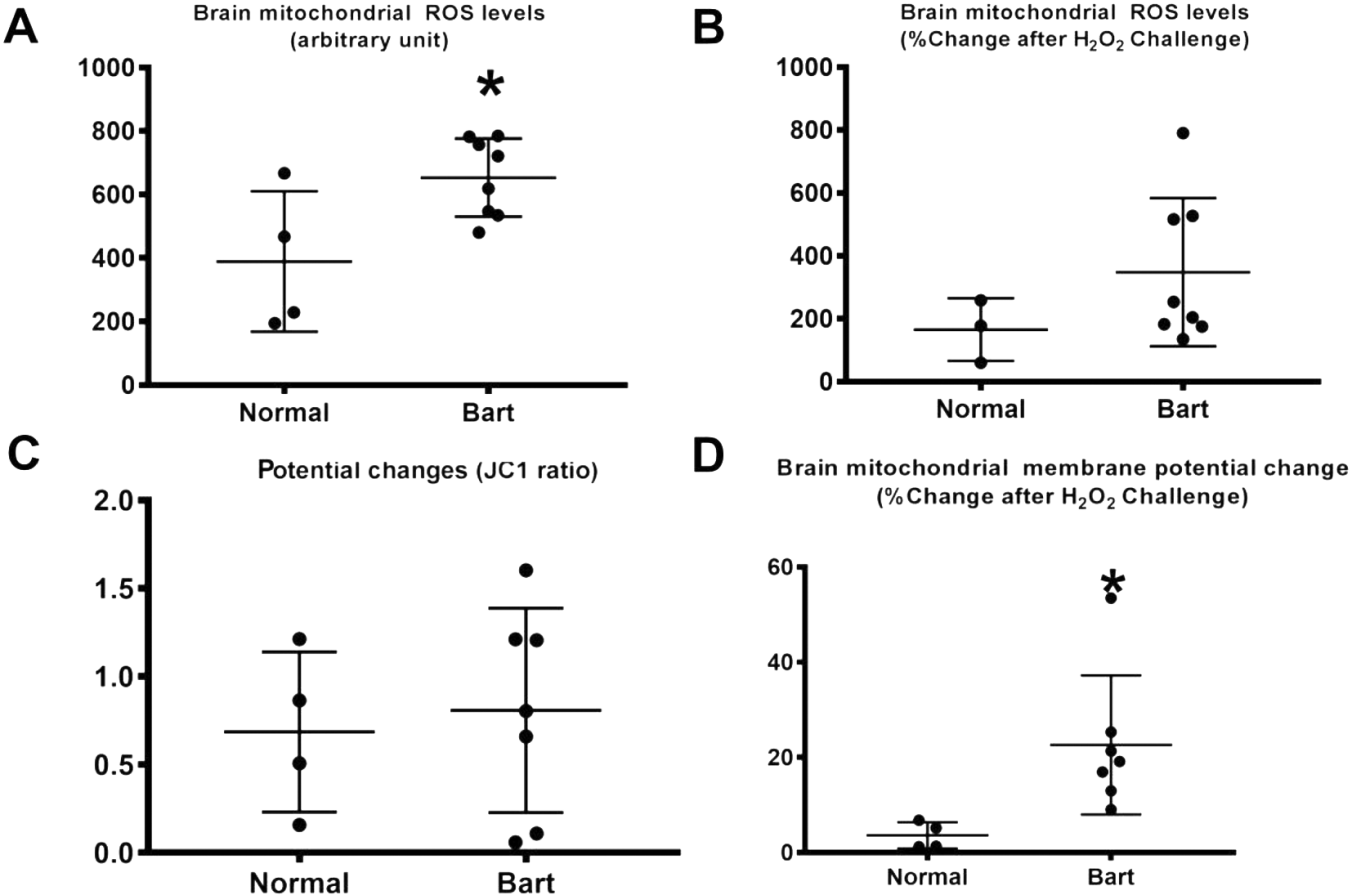
Mitochondrial dysfunction, as indicated by increased membrane potential change and ROS production, was observed in the brains of Hb Bart’s fetuses compared with those of the normal ones (*p-value < 0.001)

In the analysis of the fetal heart and brain tissue, mitochondrial dysfunction was clearly demonstrated in fetal brain tissue and in the fetal myocardium, as presented in Fig. 1. This is indicated by a significant increase in the membrane potential change and a trend to increase of ROS production level, though not reach a significant level, in the heart of the fetuses affected by Hb Bart’s disease, as presented in Fig. 2. Likewise, brain mitochondrial ROS level and membrane potential change after H_2_O challenge were significantly increased in the affected fetuses, as presented in Fig. 3. Note that the sample size was reduced in molecular study, since high quality of the sample could not be prepared or high-quality laboratory result could not be achieved in some cases, (n: 4 in normal fetuses and 7–9 in the affected fetuses).

## Discussion

### Introduction

As already known, fetuses have a high potential reserve or a compensatory mechanism to cope with anemia. However, the efficiency of this mechanism in preventing detrimental effects caused by anemia is unclear. The main purpose of response is to maintain oxygen delivery in the target organs. Therefore, the cardiovascular system works harder to increase oxygen delivery. The main objective of this study is to investigate whether the adaptive response to anemia is detrimental to the pumping machine (heart), as a consequence of increased work, and the target organ (represented by the brain), as an indication of the effectiveness of compensation in providing oxygen delivery. This objective is achieved by evaluating the cellular damages of the fetal myocardium and brain tissues along with clinical assessment of the fetal cardiovascular system.

### The main findings

In addition to increased cardiovascular work in response to fetal anemia, as indicated by increased cardiac output and enlargement, the main new insight gained from this study is that though the heart works well, as indicated by normal MPI, SF and preload index, the hypoxic insults of fetal anemia are still demonstrated in both the pumping machine (heart) and perfused organs. The findings indicate that the compensatory system to cope with long-term anemia may not perfectly work in maintaining oxygen delivery in the target organs.

### Cardiac morphological changes

Based on this study and previous studies [16–19], in response to fetal anemia, the fetuses attempt to increase cardiac output to meet the requirement of oxygen delivery in target organs. As a consequence, the fetal heart increases in size, either assessed by cardiac area, cardiac diameter or CTR, as demonstrated in this study, which is consistent with other previous studies. Cardiomegaly associated with fetal anemia is typically symmetrical, though the right side seems more pronounced in the early phase, with minimal or no hypertrophy. Of note, in spite of marked cardiac dilatation, the ventricular wall thickness had a trend to increase, but not reach a significant level, indicating that the increased heart size in fetal anemia is mainly caused by volume load rather than pressure load on the heart or eccentric hypertrophy rather than concentric hypertrophy. Additionally, the heart tended to increase more in transverse diameter than in the long axis plane, resulting in a decrease in global sphericity index.

### Functional changes

As consequences of increased cardiac output and hypervolemia, several functional changes in the cardiovascular system occur, as follows. (1) Increased cardiac output: This study demonstrated that in the affected fetuses, cardiac output significant increased compared with those of the control, indicating that fetuses with anemia are in a hyperdynamic state because of high cardiac output and hypervolemia. This finding is consistent with those of other previous studies. For example, Sirilert et al. [20] showed that in fetuses with Hb Bart’s disease, a larger blood volume crosses the foramen ovale, causing a greater septum primum excursion. (2) Myocardial performance (Tei) index (MPI): This study showed confirmatory findings that MPI is significantly increased in the affected fetuses compared with those of non-anemic fetuses. However, the functional performance is still in the normal reference ranges [7], implying that the heart works well during increased workload or has high tolerance during high-output state in early gestation. Note that both ICT and IRT were significantly prolonged in the affected fetuses, mainly responsible for an increase in MPI. The ICT tended to change earlier than the IRT, signifying that ventricular systolic function is more sensitive to fetal anemia. Different from other disorders of elevated afterload, such as fetuses of uteroplacental insufficiency, in which IRT is earlier prolonged than ICT, our findings suggest that fetal anemia may first interfere with ventricular systolic function before diastolic function. Additionally, this study provides two other pieces of evidence which indicate that the heart works well. (1) Shortening fraction: during the high output state with cardiac enlargement, the hearts of the affected fetuses still had a relatively good shortening fraction, comparable with those of non-anemic fetuses. (2) Cardiac preload index was not significantly changed in the affected fetuses, in spite of the increase in cardiac output with hypervolemia, assessed with ductus venosus Doppler ultrasound. This conclusion is based on the finding of high forward flow (positive a-wave) in the ductus venosus during atrial contractions, suggesting normal central venous pressure. The reasons why the hearts of the affected fetuses showed high tolerance in spite of increased workload due to hypervolemia and cardiac output may be because of a decrease in afterload induced by anemia [21, 22] and low blood viscosity [23].

### Brain compensation

As expected, this study confirmed the previous knowledge that MCA-PSV was significantly increased in the affected fetuses. In fetal anemia, low blood viscosity enhances the red blood cell flow, resulting in higher velocity of blood flow in all great arteries [24]. Accordingly, MCA-PSV is more commonly used, currently, as a non-invasive method in the assessment of fetal anemia in clinical practice [25], especially in the evaluation of Rh alloimmunization, fetal Hb Bart’s disease, parvovirus B19, etc. In addition to increased MCA-PSV, this study provides evidence that there was also a decrease in MCA-PI or an increase in end diastolic flow, probably caused by autoregulation to increase cerebral circulation in response to anemic hypoxia. In brief, this study suggests that there is a significant increase in cerebral blood flow in the affected fetuses, as indicated by increased cardiac output and increased MCA-PSV together with decreased MCA-PI. Nevertheless, these marked cardiovascular changes do not seem to achieve the goal of compensation in terms of oxygen delivery in the fetal brain. This conclusion is based on the fact that mitochondrial dysfunction and oxidative stress levels indicating cellular damage in fetal brain tissues were significantly higher in the affected fetuses. In other words, in spite of the compensatory mechanism of cardiovascular changes in fetal anemia, there are still residual hypoxic insults in the target organs, though cardiovascular function by clinical assessment seems normal.

### Cellular damages

Mitochondrial dysfunction is significantly higher in both the fetal myocardium and brain tissue of the fetuses affected by Hb Bart’s disease, whereas oxidative stress levels are significantly increased in brain tissues and tended to increase in cardiac tissue but not reach significant, possibly caused by a small sample size. We may conclude that hypoxic insult during fetal compensation by increasing cardiac output to meet the requirement of tissue perfusion in target organs is detrimental to both the heart and target organs, even in the early phase when the fetuses showed normal cardiac function, clinically assessed by prenatal ultrasound just before pregnancy termination. In other words, subtle cellular damage by anemic hypoxia was also not completely overcome by compensatory mechanism, raising a great concern of chronic hypoxia in all developing vital organs of the fetuses, in spite of the normal cardiac function. Accordingly, our evidence suggests that subtle but serious effects of fetal anemic hypoxia cannot be ignored. Our findings in human anemic fetuses are consistent with previous studies in different animal models, which show that fetal brain is vulnerable to oxidative stress and hypoxia, leading to impairment of neurogenesis disruption of cortical migration and mitochondrial damage and neuro-inflammation [26]. Fetal hypoxia caused by uteroplacental insufficiency or fetal growth restriction are also linked to increased oxidative stress levels, mitochondrial dysfunction [27], causing cellular damages in several organs especially the heart and brain. Likewise, fetal life evolving in a hypoxic environment, such as pre-eclampsia or type I diabetes can lead to increased free radical production and correlate with perinatal outcomes [28]. Overwhelming evidence supports that fetal chronic hypoxia secondary to fetal growth restriction is linked to an increased risk of adult cardiovascular disease in later life [1–4]. Due to the fact that our evidence, though based on a small sample size, fetal anemia hypoxia can cause cellular damage in myocardium and brain tissues in spite of adequate functional compensation indicated by fetal echocardiography, it is reasonable to hypothesize that fetal anemia, even in pre-hydropic state, can probably predispose to adult disease in later life.

### Weaknesses

The weaknesses of this study are as follows: (1) The results derived from this study should be interpreted with precaution since these results are based on fetuses affected by Hb Bart’s disease, whose natural course might be different from those of other causes of anemia. Fetuses with Hb Bart’s disease can theoretically have iron overload secondary to red cell destruction and possibly leading to myocardial cellular damage, as commonly seen in adults with severe thalassemia who are long exposed to iron overload. However, our previous study showed that fetal myocardial cellular damage in Hb Bart’s fetuses was not associated with iron overload. Therefore, convincingly, the myocardial cellular damage found in this study was directly associated with anemic hypoxia, not iron overload, and may be expected to occur in anemia of any causes. (2) The sample size of the control or non-anemic group is relatively small, due to the rarity of non-anemic fetuses having an indication for pregnancy termination, leading to a limited amount of tissues for study. (3) The varying degree of anemia might have influenced on the results.

### Strengths

The strengths of this study are as follows: (1) The results of post-abortal molecular study could represent the clinical changes of the fetuses in utero since prenatal assessment was performed just before pregnancy termination. (2) The evaluation of cellular damage due to fetal anemia was performed in both the pumping machine (heart) during increased workload, and the target organ (brain) to test the efficiency of the cardiovascular system in oxygen delivery. (3) The evidence of anemia effects was provided by both clinical changes (assessed by comprehensive prenatal ultrasound) and molecular levels.

### Conclusions

In response to anemia, fetuses attempt to increase cardiac output to meet oxygen requirement in the target organs. The cardiovascular changes to achieve the goal of providing tissue perfusion are as follows: (1) morphological changes: an increase in cardiac size, typically more pronounced in transverse diameter than in the long axis, global dilatation with minimal hypertrophy; (2) functional changes: an increase in MPI with earlier prolonged ICT and prolonged IRT, maintaining good contractility without increased preload index in spite of obviously increased cardiac output but the functions at mid-pregnancy are still within normal limits.; (3) Increased target organ circulation as evidenced by elevated MCA-PSV and a decrease in MCA-PI or increased end-diastolic flow; (4) Significant cellular damage in the pumping organ (heart) and perfused organ (brain).

## Data Availability

The data of this study are available from the corresponding authors upon request.
